# Enhancing Alzheimer’s disease diagnosis and staging: a multistage CNN framework using MRI

**DOI:** 10.3389/fpsyt.2024.1395563

**Published:** 2024-06-24

**Authors:** Muhammad Umair Ali, Kwang Su Kim, Majdi Khalid, Majed Farrash, Amad Zafar, Seung Won Lee

**Affiliations:** ^1^ Department of Artificial Intelligence and Robotics, Sejong University, Seoul, Republic of Korea; ^2^ Department of Scientific Computing, Pukyong National University, Busan, Republic of Korea; ^3^ Interdisciplinary Biology Laboratory (iBLab), Division of Biological Science, Graduate School of Science, Nagoya University, Nagoya, Japan; ^4^ Department of Computer Science and Artificial Intelligence, College of Computing, Umm Al-Qura University, Makkah, Saudi Arabia; ^5^ Department of Precision Medicine, Sungkyunkwan University School of Medicine, Suwon, Republic of Korea

**Keywords:** Alzheimer’s disease, MRI, convolutional neural network, dementia, neuroimaging

## Abstract

This study addresses the pervasive and debilitating impact of Alzheimer’s disease (AD) on individuals and society, emphasizing the crucial need for timely diagnosis. We present a multistage convolutional neural network (CNN)-based framework for AD detection and sub-classification using brain magnetic resonance imaging (MRI). After preprocessing, a 26-layer CNN model was designed to differentiate between healthy individuals and patients with dementia. After detecting dementia, the 26-layer CNN model was reutilized using the concept of transfer learning to further subclassify dementia into mild, moderate, and severe dementia. Leveraging the frozen weights of the developed CNN on correlated medical images facilitated the transfer learning process for sub-classifying dementia classes. An online AD dataset is used to verify the performance of the proposed multistage CNN-based framework. The proposed approach yielded a noteworthy accuracy of 98.24% in identifying dementia classes, whereas it achieved 99.70% accuracy in dementia subclassification. Another dataset was used to further validate the proposed framework, resulting in 100% performance. Comparative evaluations against pre-trained models and the current literature were also conducted, highlighting the usefulness and superiority of the proposed framework and presenting it as a robust and effective AD detection and subclassification method.

## 1 Introduction

Alzheimer’s disease (AD) is a progressive neurodegenerative disease that causes irreversible cognitive dysfunction, amnesia, and progressive loss of brain function, eventually resulting in an inability to function independently in daily life (1). AD is the most prevalent type of dementia and requires a high level of medical attention. Global projections anticipate a significant increase in the number of individuals affected by AD, with an estimated 152 million affected by 2050 compared to the current 47 million. This poses challenges across the economic, medical, and societal domains (2). Every 3 seconds, one person worldwide is affected by dementia, with AD accounting for 60% of all dementia cases (3). The phases of dementia associated with AD can be broadly divided into the following categories: i) mild cognitive impairment (MCI), ii) mild dementia, iii) moderate dementia, and iv) severe dementia. MCI, which is often characterized by memory loss with increasing age, can lead to dementia in certain individuals. Individuals with mild dementia occasionally struggle with cognitive deficits that affect their daily activities. The symptoms include disorientation, memory loss, uncertainty, personality changes, and difficulties in performing daily chores. Moderate dementia significantly complicates daily life and requires greater assistance and care. These symptoms are more prominent and resemble those observed in patients with mild dementia. Individuals may require assistance with basic tasks, such as brushing their hair, and experience significant personality changes, including sudden onset of irritation or anxiety. Sleep disturbances were also frequent. Patients with severe dementia experience a marked decline in their condition as symptoms progress. Loss of communication skills may necessitate full-time care. The inability to perform basic activities, such as sitting in a chair or holding one’s head up, and loss of bladder control are characteristics of this stage.

Currently, there are no viable treatments to cure or decrease the progression of AD, and a complete understanding of its pathogenesis remains elusive. MCI is the transitional stage between AD and normal cognitive aging. Individuals with MCI are more likely to develop AD than those with age-matched healthy cognition (4). Preventive strategies to limit disease progression as well as efficient treatment and care procedures depend on the early detection of AD.

Medical history, physical examination, and further diagnostic tests such as neurological screenings that examine reflexes, coordination, and muscle tone are all common components of AD diagnostic evaluation (5). Magnetic resonance imaging (MRI), computed tomography (CT), and positron emission tomography (PET) are important imaging techniques for the diagnosis of AD. In particular, fluid-attenuated inversion recovery sequences in MRI are used to suppress the cerebrospinal fluid, allowing for a more thorough evaluation of anatomical structures and examination of gliotic alterations (6, 7). The advantages of MRI include improved flexibility, clear tissue contrast, lack of ionizing radiation, and the capacity to provide insightful information regarding the structure of the human brain (8). Developing an improved computer-aided diagnostic system capable of analyzing MRI images to determine whether individuals have AD or are in good health is crucial.

A wide range of machine-learning methodologies that integrate neuroimaging have improved the accuracy of identifying distinct dementia subtypes (9). Conventional machine-learning methods such as support vector machine (SVM) (10), random forest (11), and linear program boosting algorithms (12) have been used to detect AD using MRI (13). Furthermore, the variants of SVM and the ensemble of classifiers were also proposed. However, conventional machine-learning approaches frequently require the manual selection of predefined brain areas of interest based on established MRI markers linked to AD. Due to an inadequate understanding of definitive MRI biomarkers for AD, predetermined regions are likely unable to include all the information required to unravel the complexities of AD. In addition to being labor-intensive and time-consuming, manual selection also has the potential for subjective errors. Likewise, when it comes to choosing ensemble methods, managing computational expenses poses a significant challenge.

In contrast, deep-learning networks adopt a more advanced approach, including methods such as convolutional neural networks (CNNs), deep belief networks, recurrent neural networks, long-short term memory, stacked autoencoders, and restricted Boltzmann machine (14–17). These techniques combine low-level features in the data to automatically create a higher-level, more abstract representation of a learning system (18). Because of high image classification accuracy, deep learning state-of-the-art techniques are preferred over conventional machine-learning methods. In general, CNN and its variants are the most popular deep-learning algorithms due to their better performance in identifying AD. CNN models are widely used in segmentation, object recognition, and classification (19–21). This popularity can be attributed to several benefits including leveraging the spatial information of neighboring pixels, direct acceptance of image data as input, and efficient reduction of model parameters by utilizing weight-sharing, subsampling, and local receptive fields. A CNN trained using MRI slices can automatically extract features from images, thereby eliminating the requirement for manual feature selection during the learning phase (22). Furthermore, they also display higher generalization skills when dealing with scans from various sources or scanners.

Several CNN models have recently been proposed as diagnostic tools (23). Lu et al. (24) presented a multimodal CNN model using MRI and PET. Their proposed framework yielded an accuracy of 82.4% for patients with MCI who were subsequently diagnosed with AD. The model achieved a classification accuracy of 86.3% for individuals without dementia. In another study (25), accuracies of 90.05 and 85.55% were achieved for different datasets using CNN-based features and softmax as classifiers for binary classification (normal controls and AD). A pretrained AlexNet CNN model was used to retrieve deep features, and conventional machine-learning methods were used as classifiers (26). The results showed that the proposed methodology outperformed other handcrafted features with an accuracy of 99.21%. A VGG-16-based CNN model was used to classify the MRI slices (27). The model achieved a high accuracy of 95.73% for tertiary classification problems (early MCI, normal control, and late MCI). Pan et al. (28) hybridized ensemble learning and CNN models to classify brain MRI for various classification problems. Their models exhibited reasonable classification performance. Murugan et al. (29) presented the DEMNET model to classify various stages of dementia using MRI. They used the synthetic minority oversampling technique (SMOTE) approach to resolve class imbalance issues. Their model achieved a high accuracy of 95.23% for four classes. Although DEMNET shows high classification performance, the accuracy of an augmented dataset is not considered reliable for real-time applications. Recently, Fathi et al. (30) introduced a weighted probability-based ensemble method to combine six 2D-CNN architectures and obtain a high classification rate of 93.88 for four classes. Furthermore, they compared different ensemble methods and showed that the ensemble methods yielded better results than individual architectures. Kang et al. (31) proposed a three-round learning strategy based on a 3D deep convolutional generative adversarial network model and obtained an accuracy of 92.8% for two classes.

To improve the prediction performance, numerous studies have recently included attention models. The attention models focus on the most informative image regions. By combining two distinct attention modules (i.e., enhanced non-local attention and coordinate attention), Illakiya et al. (32) presented an adaptive hybrid attention network to enhance the performance of the DenseNet architecture, resulting in a higher classification accuracy of 98.53%. Similarly, in another study (33), an integrated model consisting of a depthwise group shuffle, global context network, hybrid multi-focus attention block, and EfficientNEt-B0 was developed to improve the prediction performance of MCI classification. Zhang et al. (34) developed an end-to-end 3D CNN framework based on the ResNet architecture, which employs multi-modality brain images to perform AD diagnostic and MCI prediction tasks by integrating 3D attention processes with multi-layer feature fusion algorithms. They showed that their multimodal model outperformed a single modality in predicting AD and MCI, with superior results of 6.37% and 3.51%, respectively. Some studies have also combined the transformer and attention networks. Hu et al. (35) designed a classifier model by combining a CNN with a swine transformer. In addition, they added a shift window attention mechanism to the transformer to improve the feature extraction. They achieved an accuracy of 93.5% for the two classes using their proposed model. Illakiya et al. (36) utilized a swine transformer, a dimension-centric proximity-aware attention network, and an age deviation factor to improve feature extraction from brain MRI images. The proposed network improves the classification results by utilizing a novel feature fusion strategy that incorporates global, local, and proximal characteristics, as well as dimensional dependencies. The literature describes various methods for classifying AD using conventional machine-learning and deep-learning models. However, there are challenges related to the large number of model parameters, training time, and high performance without augmentation in multiclass AD classification.

To address these issues, the primary contributions and steps of this study are outlined below:

- We hypothesized that leveraging the frozen weights of the developed CNN on correlated medical images facilitated the transfer learning process for sub-classifying dementia classes.
- To prove the hypothesis, a lightweight CNN model was developed in stage 1 to detect dementia using MRI images after preprocessing.
- In stage 2, a new model was built by reutilizing the frozen weight of the developed model for further classification of dementia into mild, moderate, and very mild dementia using transfer learning.
- Various online AD datasets were used to validate the proposed model.
- Various pre-trained models were trained using the same parameters and datasets for a fair comparison.

The results were compared with those reported in the literature.

## 2 Materials and methods

### 2.1 Proposed framework

**Figure 1:**
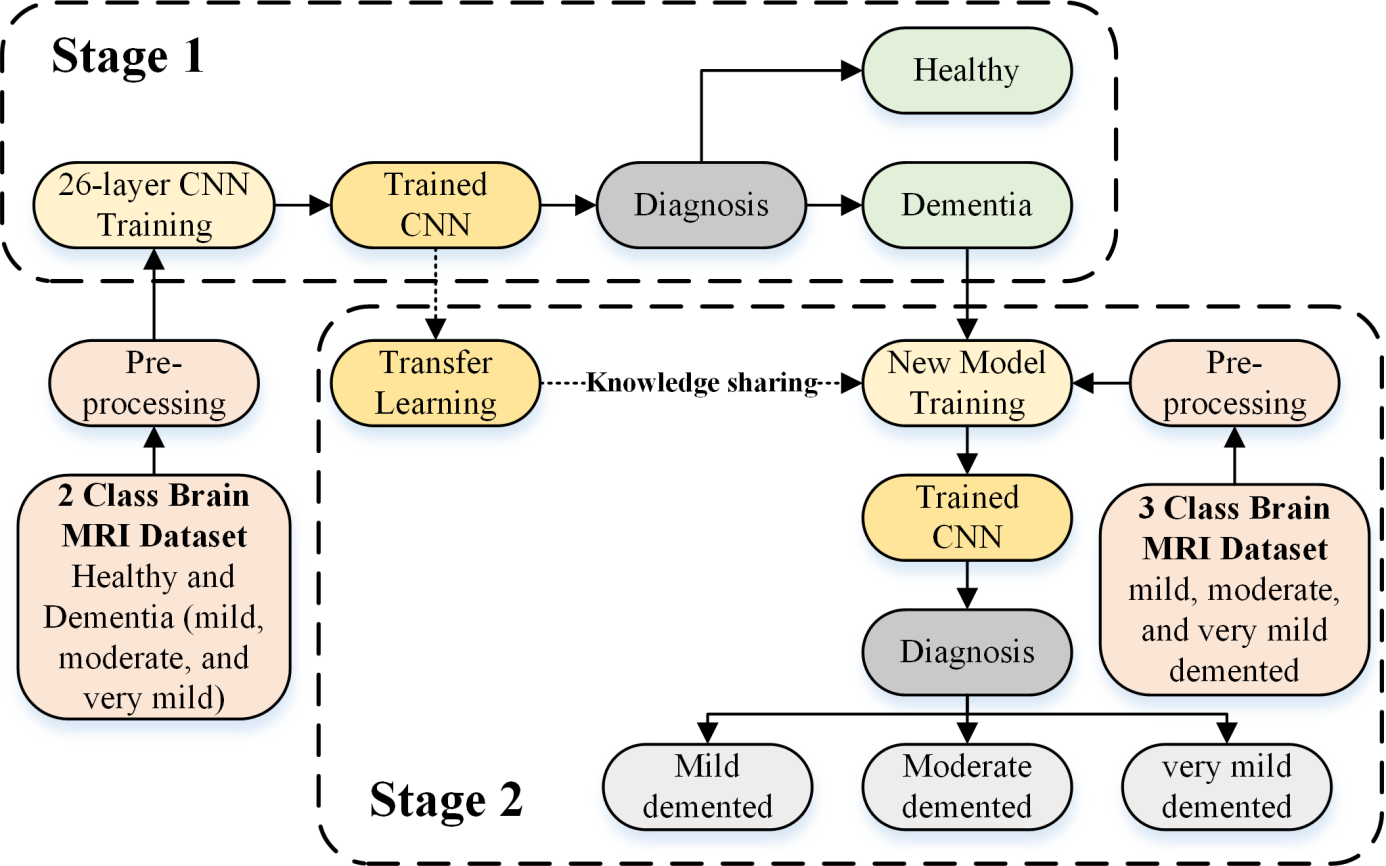
CNN-based proposed framework for dementia detection and subclassification.

The proposed CNN-based framework is depicted in **Figure 1**. In the proposed framework, dementia detection and sub-classification were divided into two stages (i.e., stage 1 and stage 2). In stage 1, the brain MRI scans were classified into two classes (healthy and dementia) using a proposed 26-layer CNN model (presented in Section 2.4). In stage 2, transfer learning was used to reutilize the frozen weights of the 26-layer CNN model (i.e., developed in stage 1) to fine-tune the new transfer-learned model by replacing the last three layers of the developed CNN for dementia subclassification (mild, moderate, and very mild dementia).

### 2.2 Datasets

Two different datasets (the Alzheimer’s dataset (four classes of images) and ADNI_Extracted_Axial) were used to validate the proposed approach. Both datasets are publicly available (https://www.kaggle.com/datasets/tourist55/alzheimers-dataset-4-class-of-images and https://www.kaggle.com/datasets/katalniraj/adni-extracted-axial; accessed November 13, 2023). The specifications of this dataset are listed in **Table 1**.

**Table 1:** Details of various online available datasets.

Dataset	Classification	Subclassification	MRI Slices	No. of Samples
Alzheimer’s Dataset	Healthy	Lack of Dementia	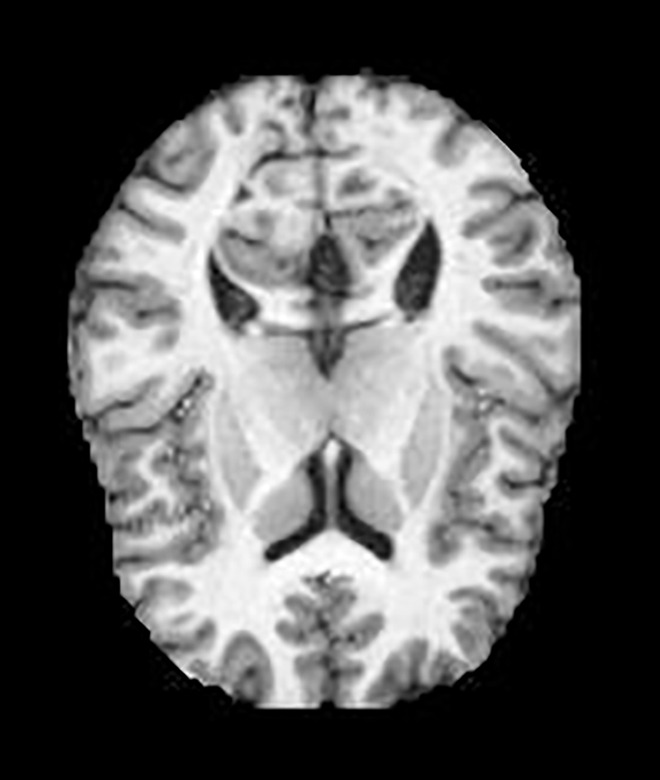	2560
	Dementia	Mild Dementia	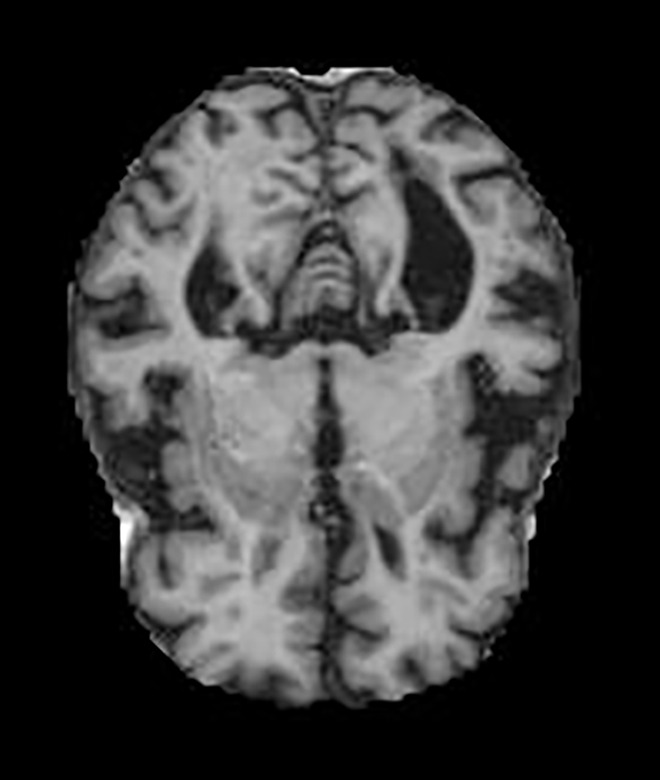	717
		Moderate Dementia	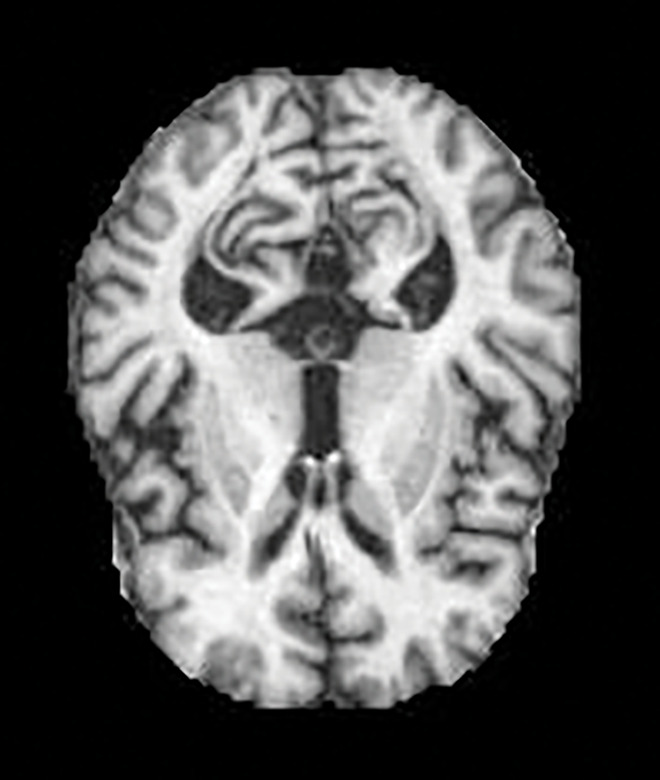	52
		Very Mild Dementia	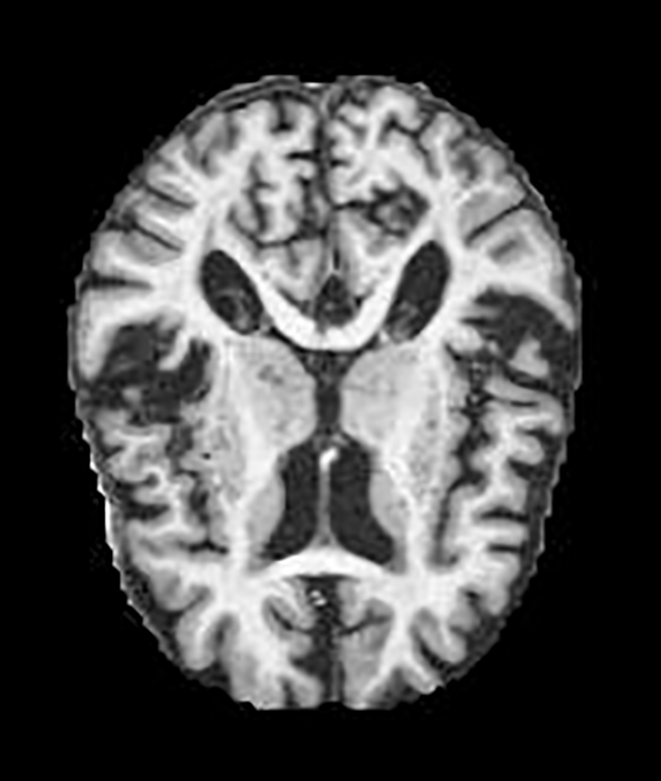	1792
ADNI_Extracted _Axial	Healthy	Common Normal	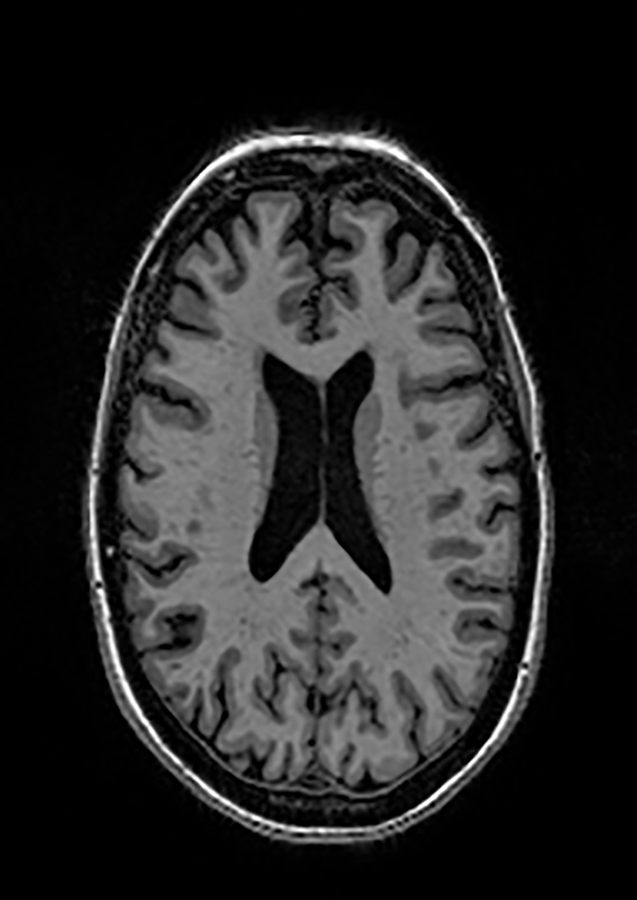	1440
	Dementia	Alzheimer’s Disease	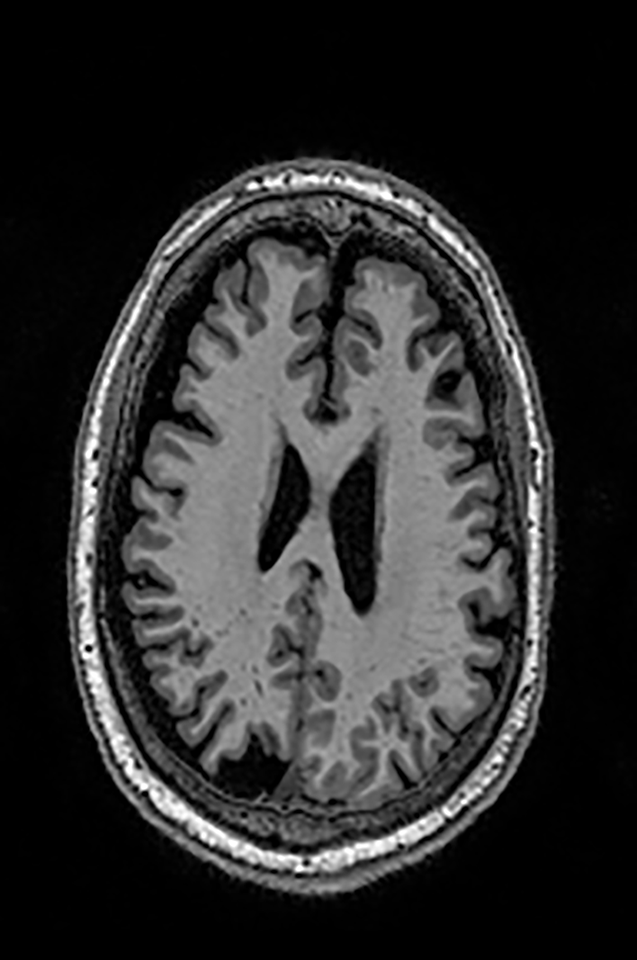	1124
		Mild Cognitive Impairment	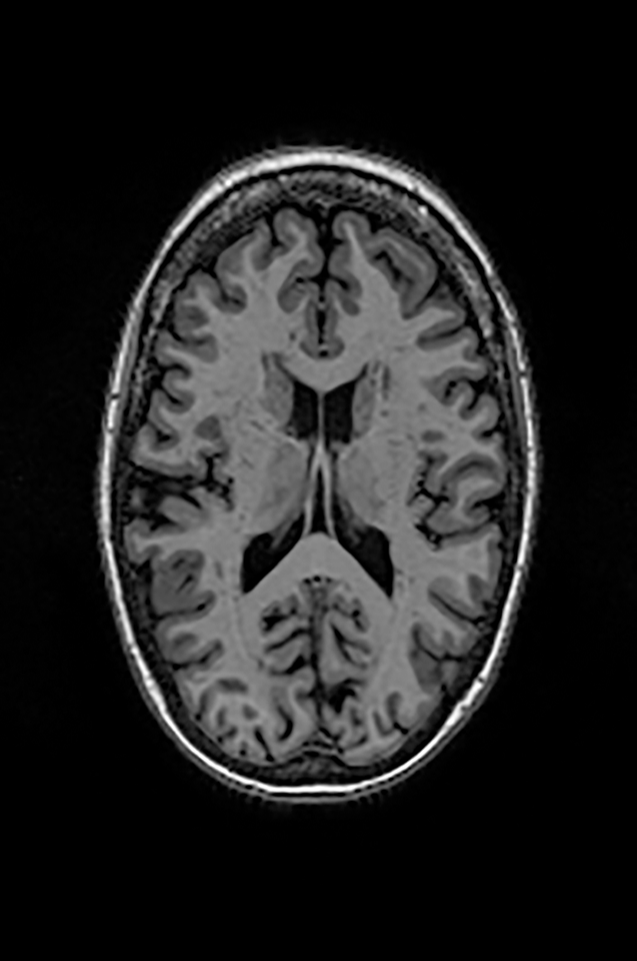	2590

### 2.3 Pre-processing

In CNN applications, irrelevant information in an image can adversely affect the subsequent image-processing steps. Preprocessing is imperative to address these issues and ensure the accuracy of subsequent steps in image processing. Therefore, a cropping and zero-center approach was applied to remove unwanted information and normalization (37). After preprocessing and normalization of the dataset, the images were input into a developed CNN, which identified the AD-affected area by extracting discriminating features.

### 2.4 Design of CNN for AD Detection

CNNs are a type of deep-learning model developed specifically for analyzing structured grid data, such as images. Their ability to learn hierarchical feature representations autonomously has revolutionized computer vision tasks. CNNs comprise layers that perform convolutional operations to capture local patterns and pooling operations to reduce spatial dimensions. Weight sharing characterizes these networks, allowing them to recognize comparable features across the input space. Convolutional layers are often followed by fully connected layers to achieve a high level of feature integration and classification. CNNs excel at image identification, object detection, and segmentation and demonstrate superior performance across various domains. The ability of CNNs to automatically extract significant characteristics from raw data makes them valuable tools for complicated pattern detection, leading to the development of artificial intelligence.

In this study, a 26-layer CNN model was developed from scratch to detect dementia in stage 1. This architecture comprised five blocks of convolutional layers, batch normalization, ReLU activation functions, and max-pooling layers. Fully connected layers and a softmax output layer were used for the classification. The model uses dropout for regularization, and the final output layer employs cross-entropy loss. Complete information regarding the designed CNN model is presented in **Table 2**. The details of the layers are presented in the subsequent sections.

**Table 2:** Details of the designed CNN model for AD detection.

Layer No.	Type of Layer	Properties
1	Image Input	227×227×1 images with ‘zerocenter’ normalization
2	2-D Convolution	32 3×3×1 convolutions with stride [2 2] and padding [0 0 0 0]
3	Batch Normalization	Batch normalization with 32 channels
4	ReLU	ReLU
5	2-D Max Pooling	2×2 max pooling with stride [2 2] and padding [0 0 0 0]
6	2-D Convolution	32 3×3×32 convolutions with stride [1 1] and padding [2 2 2 2]
7	Batch Normalization	Batch normalization with 32 channels
8	ReLU	ReLU
9	2-D Max Pooling	2×2 max pooling with stride [2 2] and padding [0 0 0 0]
10	Batch Normalization	Batch normalization with 32 channels
11	2-D Convolution	64 3×3×32 convolutions with stride [1 1] and padding [2 2 2 2]
12	ReLU	ReLU
13	2-D Max Pooling	2×2 max pooling with stride [2 2] and padding [0 0 0 0]
14	2-D Convolution	128 3×3×64 convolutions with stride [1 1] and padding [2 2 2 2]
15	Batch Normalization	Batch normalization with 128 channels
16	ReLU	ReLU
17	2-D Max Pooling	2×2 max pooling with stride [2 2] and padding [0 0 0 0]
18	2-D Convolution	256 3×3×128 convolutions with stride [1 1] and padding [2 2 2 2]
19	Batch Normalization	Batch normalization with 256 channels
20	ReLU	ReLU
21	Batch Normalization	Batch normalization with 256 channels
22	Fully Connected	1024 fully connected layer
23	Dropout	30% dropout
24	Fully Connected	2 fully connected layer
25	Softmax	–
26	Classification Output	–

CNN, convolutional neural network; AD, Alzheimer’s disease; ReLU, rectified linear unit activation.

#### 2.4.1 Input layer

The input layer of the developed model represents the initial layer and accepts normalized images. This layer sets the input size and normalization strategy for the subsequent processing.

#### 2.4.2 Convolutional layer

The foundation of any CNN model is comprised of convolutional layers. Convolutional layers are the core layers of any CNN model and are responsible for the extensive computational work. The input image is passed through this layer to produce a feature map or response by convolving it with weight filters and adding bias values. Subsequently, the feature response is passed through the following layers. Mathematically, convolution involves taking the element-wise product of the filter and a patch of the input and summing up all these products. The input (*x*), can be expressed as Equation (1).


(1) $$y=\sum W*x+b_{i}$$

where *W* and *b_i_* are the filter and bias of each filter, respectively.

#### 2.4.3 Batch normalization

Batch normalization was applied to the output of the convolutional layer. This layer normalizes the activation and enhances convergence and training stability. This introduced learnable parameters for scaling and shifting. *x* is assumed to be the convolutional layer output. Batch normalization normalizes *x* across batch dimensions using Equation (2) (38):


(2) $$\hat{x}=\frac{x-\mu }{\sqrt{\sigma^{2}+\in }}$$

where *μ* is the mean, *σ* is the variance, and ∈ is a small constant used to avoid division by zero. It scales and shifts the normalized output $y=\alpha \hat{x}+\beta$ , where *α* and *β* are learnable parameters.

#### 2.4.4 Rectified linear unit activation

The rectified linear unit (ReLU) activation function was applied element-wise. ReLUs introduce nonlinearity, which allows the model to capture more complex data patterns. If the input value is positive, the ReLU activation function immediately outputs the value. If not positive, it outputs zero. This can be mathematically expressed as Equation (3) (29):


(3) f(x)=max(0,x)

#### 2.4.5 Max pooling

Pooling layers were used between the convolutional layers to reduce the representation in the spatial domain and computation space. Max pooling helps retain essential information while reducing the computational complexity by reducing the spatial dimensions. Max pooling was calculated using Equation (4) (29):


(4) $$\text{Max Pooling}=Floor\left(\frac{\text{Input }x-\text{Pooling window size}}{\text{stride}}+1\right)$$

#### 2.4.6 Fully connected layer

The fully connected layer is densely connected. It captures high-level features from convolutional layers and prepares a classification model. Mathematically, this can be expressed as Equation (5).


(5) $$y=W*x+b_{i}$$

#### 2.4.7 Dropout layer

Dropout prevents overfitting by randomly deactivating neurons during training, enhancing model generalization.

#### 2.4.8 Softmax

Softmax applies an activation function to convert logs into class probabilities. Softmax ensures that the sum of probabilities for all classes is one.

#### 2.4.9 Classification output

It uses cross-entropy loss, specifically ‘crossentropyex’ in MATLAB, for model training. Cross-entropy measures the dissimilarity between predicted and actual class probabilities as Equation (6).


(6) $$H(y,p)=\sum_{i=1}^{n}p_{i}\cdot \log\left(y_{i}\right)$$

where *H* denotes the cross-entropy loss, *y_i_* denotes the predicted probability distribution, and *p_i_* denotes the true probability distribution.

### 2.5 Design of transfer learned CNN for subclassification

Transfer learning, a machine-learning technique, leverages pre-existing models to expedite learning in new tasks. That is, the model developed for a task is reused as the starting point for the model for a second task. This approach is particularly useful when data are scarce. The main concept of transfer learning is to leverage the features learned from tasks with a large amount of available data to improve the performance of tasks with less data. This is based on the idea that tasks share commonalities that can be utilized to improve performance. Assume that a domain is composed of two elements (39, 40), as shown in Equation (7).


(7) $$A_{m}=Y+prob(y)$$

In Equation (7), *Y* and *prob*(*y*) denote the feature space and marginal probability, respectively. Suppose a task has two components:


(8) $$L_{r}=X+\alpha$$

In Equation (8), *X* and *α* symbolize the label space and the objective function, respectively. Here, $A_{m}^{s}$ and $L_{r}^{s}$ represent the source domain and task, respectively, and $A_{m}^{t}$ and $L_{r}^{t}$ represent the target domain and task, respectively. The goal of transfer learning is to utilize knowledge from the source domain to understand the conditional probability in the target domain.

**Figure 2:**
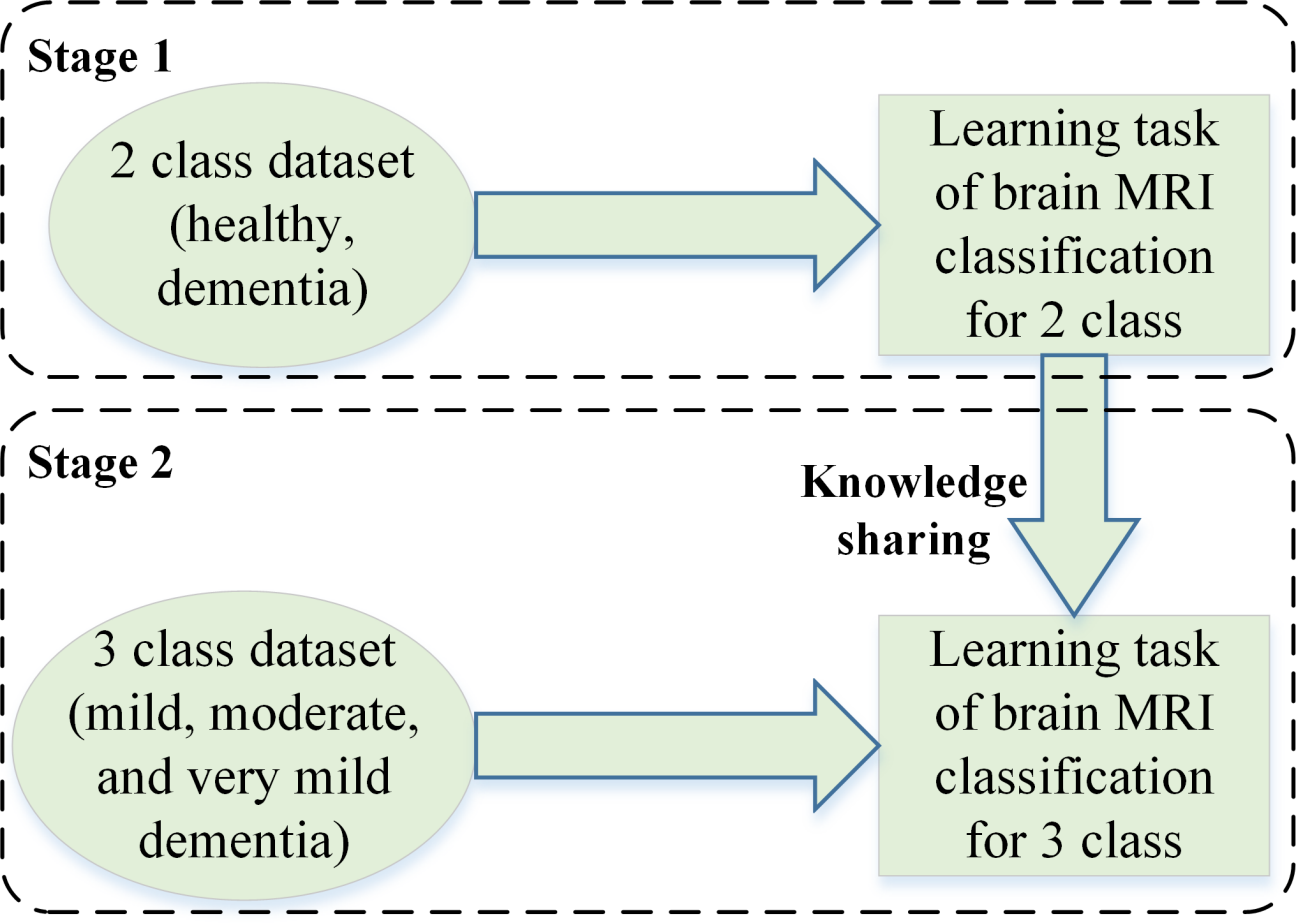
The concept of knowledge sharing.

In stage 2 of this study, transfer learning is applied to the 26-layer CNN model that was developed in the first stage (section 2.4). Reutilizing the frozen weights of the 26-layer CNN model, the model was retrained by replacing the last three layers (i.e., fully connected, softmax, and classification layers) for dementia subclassification (mild, moderate, and very mild dementia). **Figure 2** shows the concept of knowledge sharing from dementia to dementia subclassification.

## 3 Results

MATLAB 2023a was used to perform all simulations and analyses on a personal computer with the following specifications: Core i7, 12th Generation, 32 GB RAM, NVIDIA GeForce RTX 3050, 1 TB SSD, and 64-bit Windows 11 operating system. The dataset was randomly divided into 80 and 20 ratios for model training and testing, respectively. The images used for model testing were not used to train the CNN. The following initial parameters were utilized: 100 Epochs, 0.9 momentum, 128 mini batch-size, and 0.001 learning rate. The stochastic gradient descent with momentum (SGDM) solver was utilized to train and test the model.

**Figure 3:**
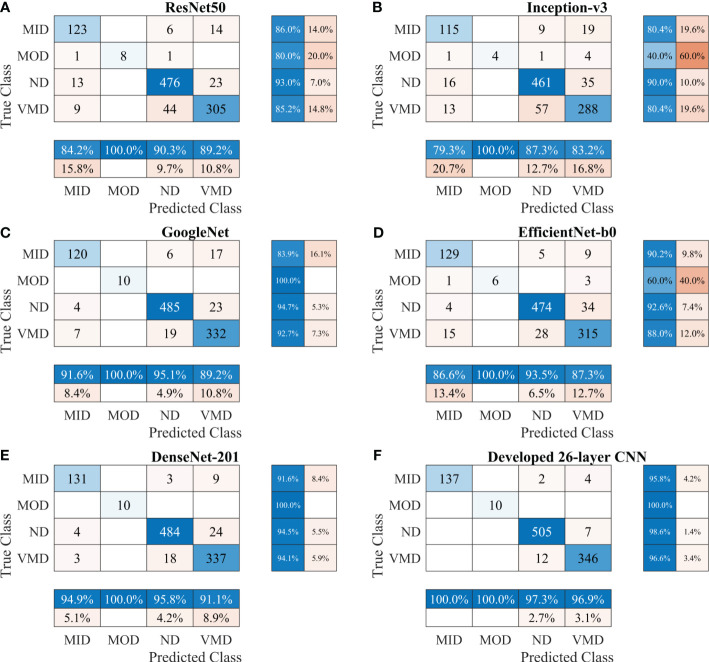
Confusion matrix of various CNN models for Alzheimer’s Dataset. **(A)** ResNet50, **(B)** Inception-v3, **(C)** GoogleNet, **(D)** EfficientNet-b0, **(E)** DenseNet-201, and **(F)** Developed 26-layer CNN. MID, mild dementia; MOD, moderate dementia; ND, non-dementia; VMD, very mild dementia; CNN convolutional neural networks.

First, various publicly available pre-trained CNNs, such as ResNet50, Inception-v3, GoogleNet, EfficientNet-b0, and DenseNet-201, were used to categorize the brain MRI dataset. Subsequently, the proposed CNN model was trained to classify the brain MRI scans using the same parameters. **Table 3** shows a performance comparison of various pre-trained models with the developed 26-layer CNN; and the confusion matrix of all models is presented in **Figure 3**, which also shows the true positive rate (TPR), false negative rate (FNR), positive predictive value (PPV), and false discovery rate (FDR).

**Table 3:** Performance comparison of various CNN models for Alzheimer’s Dataset.

Parameters	CNN					
	ResNet50	Inception-v3	GoogleNet	EfficientNet-b0	DenseNet-201	Developed 26-layer CNN
**Training Accuracy (%)**	100	100	100	100	100	100
**Training Loss**	1.9 ×10^-04^	4.3 ×10^-04^	3.6 ×10^-04^	2.8 ×10^-03^	1.4 ×10^-04^	1.8 ×10^-04^
**Validation Accuracy (%)**	88.95	84.84	92.57	90.32	93.93	97.45
**Validation Loss**	0.3938	0.5598	0.3584	0.3030	0.2152	0.07675
**Training Time**	299 min 40 s	435 min 47 s	40 min 30 s	329 min 45 s	1062 min 20 s	8 min 53 s

CNN, convolutional neural network.

**Figure 4:**
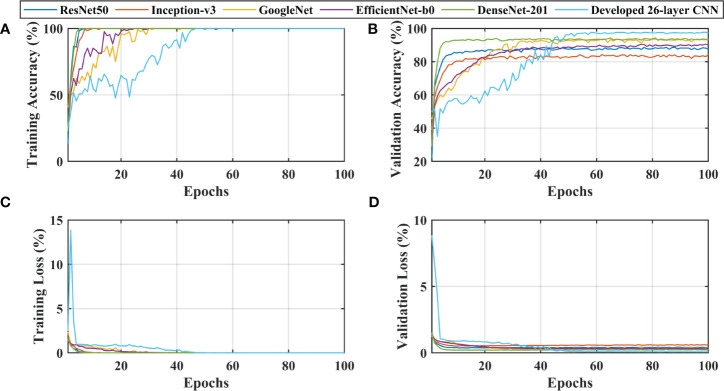
Learning curves of various convolutional neural networks. **(A, C)** shows the training accuracy and loss curves, **(B, D)** of training and depict the validation accuracy and loss curves.

After thoroughly analyzing the results presented in **Table 3** and **Figure 3**, it was found that the developed 26-layer CNN model has the best classification rate, with minimal training time and a high true positive rate for each class compared to all other pre-trained models. The learning curves of the various pre-trained models with the developed multistage 26-layer CNN are presented in **Figure 4**.

After a comprehensive analysis of the results presented in **Figure 4A**, it was found that DenseNet-201 was 100% trained in fewer epochs than ResNet50, Inception-v3, GoogleNet, EfficientNet-b0, and 26-layer CNN; it took approximately 5 epochs to stabilize the results. DenseNet-201 also demonstrates the highest validation accuracy of 93.93% among all pre-trained models for dementia classification (**Figure 4B**). In contrast, GoogleNet took almost 32 epochs to train the model and also showed a reasonable validation performance (92.57%) than the remaining pre-trained models. The developed 26-layer CNN took almost 47 epochs to reach 100% training accuracy but had the best validation accuracy of 97.45% for dementia classification. Furthermore, the time consumed for the training of the developed 26-layer CNN was only 8 min 57 s for 100 epochs, which was the fastest among all methods. It validates the robustness and high classification performance of the developed CNN model compared to other pre-trained models.

**Figure 5:**
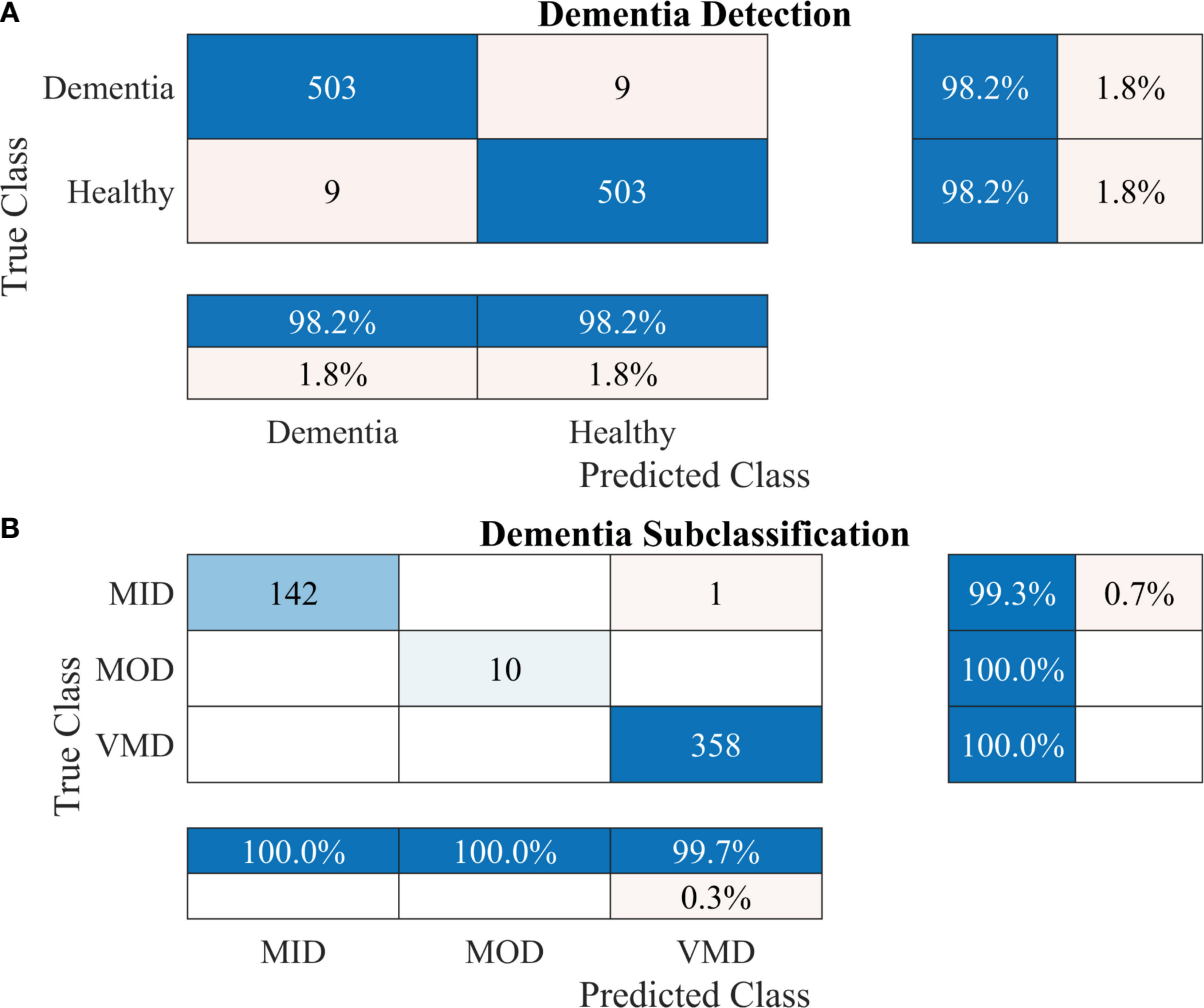
Confusion matrices using the proposed 2-stage framework. **(A)** Dementia detection and **(B)** dementia subclassification.

To further enhance the dementia detection rate and subclassification performance, the proposed framework is divided into two stages. The performance of the proposed framework for both stages is presented in **Table 4**. **Figures 5A, B** show the confusion matrix for dementia detection and sub-classification, respectively.

**Table 4:** Performance of proposed CNN-based framework for dementia detection and subclassification.

Parameters	Developed 26-layer CNN for Binary Classification	Developed Transfer Learned 26-layer CNN for Subclassification
**Training Accuracy (%)**	100	100
**Training Loss**	2.2 ×10^-04^	5.9 ×10^-04^
**Validation Accuracy (%)**	98.24	99.70
**Validation Loss**	0.0553	0.0134
**Training Time**	6 min 42 s	3 min 9 s

CNN, convolutional neural network.

**Figure 6:**
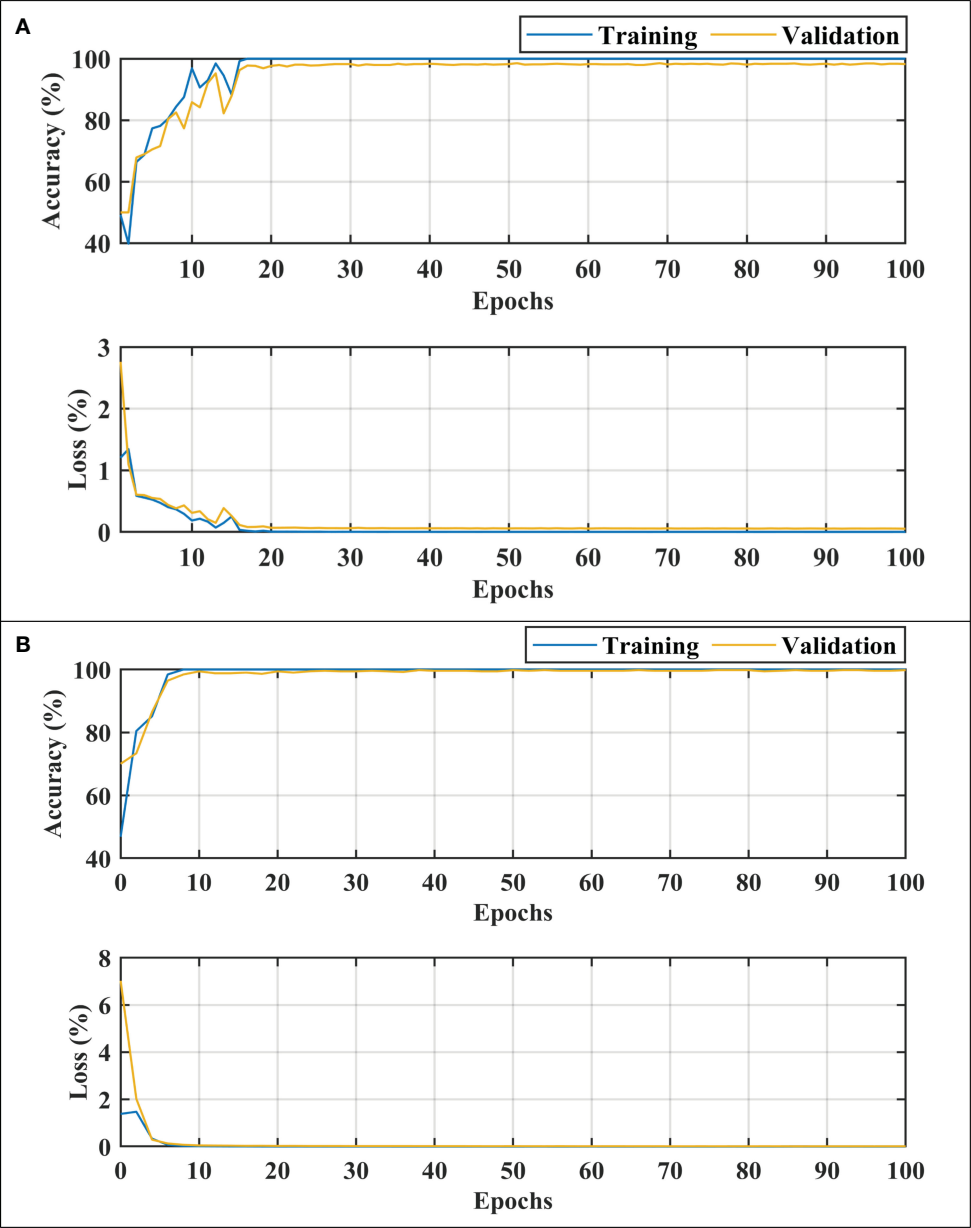
Training and loss curves. **(A)** for dementia detection and **(B)** for dementia subclassification. MID, mild dementia; MOD, moderate dementia; VMD, very mild dementia.

After carefully evaluating the result presented in **Table 4**, it was found that the proposed 2-stage approach performs efficiently to differentiate between healthy and dementia persons with a high classification rate of 98.24% with only 6 min 42 s training time. The comprehensive performance of the model is presented in **Figure 5** using a confusion matrix. The proposed model only misclassified 9 samples of each class (**Figure 5A**), resulting in a high true positive rate of 98.2% for each class. After that, a new transfer-learned model was used for the subclassification of the dementia class and yielded a high accuracy of 99.7%, with only one sample of mild dementia misclassified, as shown in **Figure 5B**. The learning curves of the dementia detection and subclassification are shown in **Figures 6A, B**, respectively.

**Figure 7:**
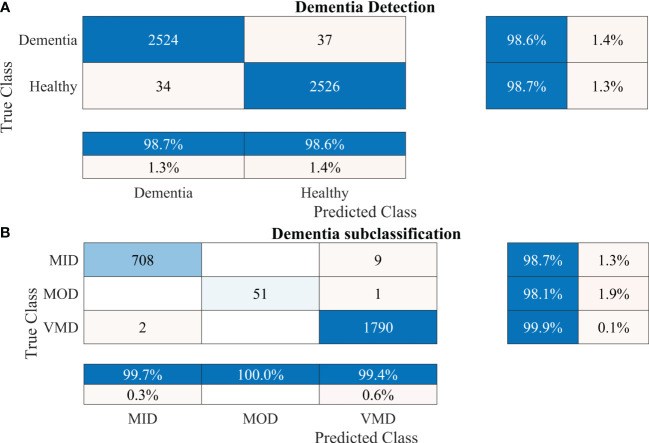
Confusion matrices using 10-fold cross-validation for the proposed 2-stage framework. **(A)** Dementia detection and **(B)** dementia subclassification. AD, Alzheimer’s disease; CI, mild cognitive impairment.

To further validate the performance of the proposed 2-stage approach against overfitting, the results of 10-fold cross-validation are presented in **Figure 7**.

**Figure 8:**
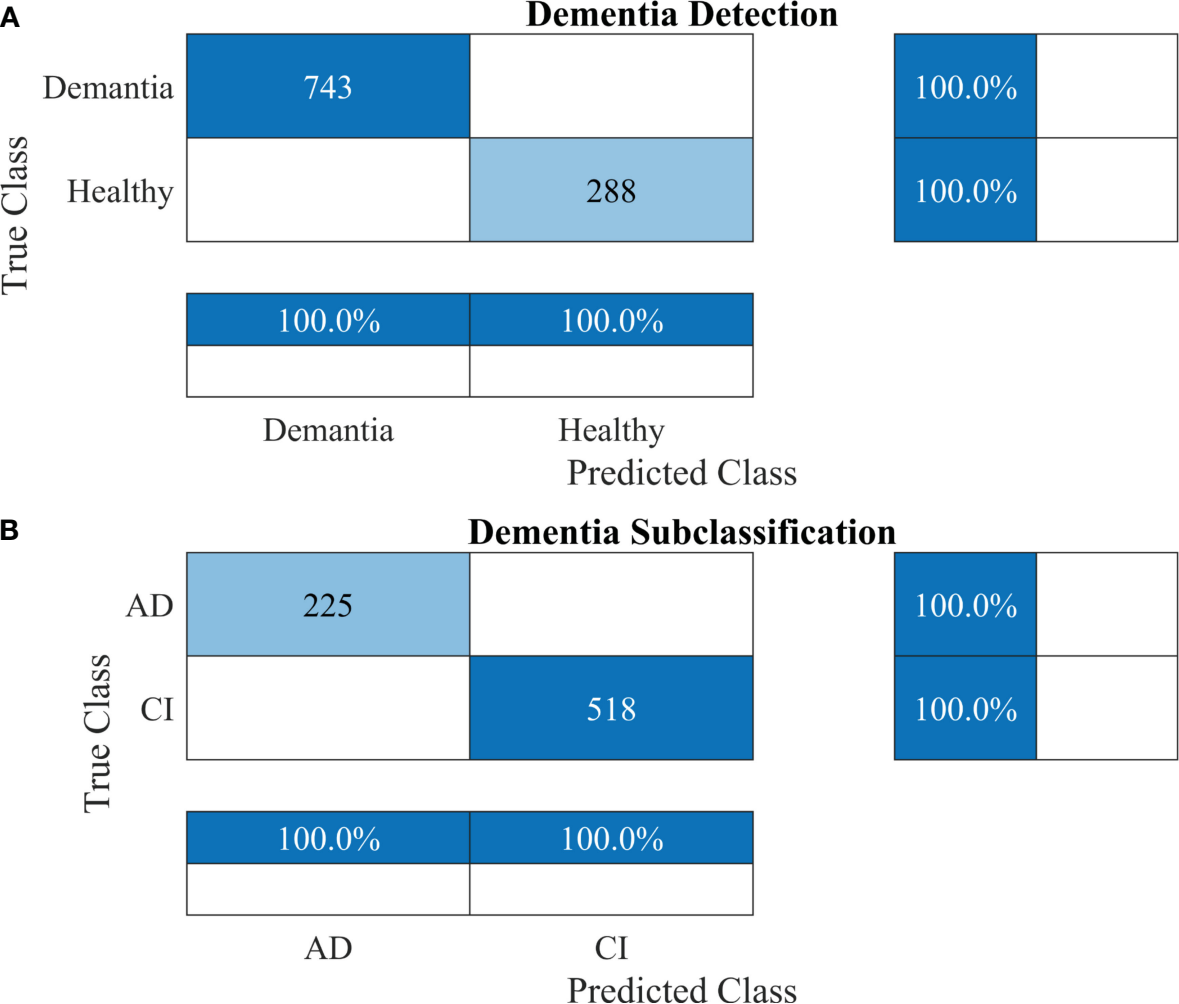
Confusion matrices using the proposed 2-stage framework for another dataset. **(A)** Dementia detection and **(B)** dementia subclassification. AD, Alzheimer’s disease; CI, mild cognitive impairment.

Furthermore, other data were also used to validate the approach’s reliability, adaptability, and accuracy. The results of dementia detection and subclassification are presented in **Figures 8A, B**, respectively.

## 4 Discussion

This study investigated the application of CNNs to identify AD and differentiate between the different stages of dementia by analyzing MRI. Recently, there has been an increase in the use of computer-aided systems for early AD detection, using both machine learning and CNNs. This study contributes to the development of an automated AD detection system for improving the operating efficiency of medical centers.

The ablation study was performed for the layers selection of developed CNN. The effect of changing the number of layers (from 22 to 34 layers) is reported in **Table 5**. All models attain 100% training accuracy showing that the deep network models match the training data closely. However, 26-layer CNN yielded the less training loss. Furthermore, the 26-layer CNN model also gives higher validation accuracy and lower validation loss as compared to other models. This shows that the 26-layer model seems to provide a greater generalization of the unknown validation data. The 26-layer CNN model was chosen because it appears to achieve an optimal balance between model complexity and generalization at this depth. Too few or too many layers may result in suboptimal performance on validation data.

**Table 5:** Results of ablation study for selection of layers.

Parameters	Developed CNNs			
	22-Layer	26-Layer	30-Layer	34-Layer
Training Loss	1.7 × 10^-3^	1.8 ×10^-04^	3.5 ×10^-04^	6.2 ×10^-04^
Training Accuracy (%)	100	100	100	100
Validation Loss	0.10741	0.07675	0.10735	0.17687
Validation Accuracy (%)	96.87	97.45	96.08	94.13
Training Time	7 min 19 s	8 min 53 s	9 min 20 s	9 min 56 s

CNN, convolutional neural network.

The developed 26-layer CNN model achieved an impressive classification accuracy of 97.45% for directly categorizing MRI scans into four classes, demonstrating superior performance with minimal training time compared to several pre-trained CNNs (**Table 3**). DenseNet-201 yielded better results than the other pre-trained networks. In the validation, the proposed model correctly classified 998 of the 1023 images (see **Figure 3F**). The TPR of all classes was higher than 95%, with a very low FDR. All pre-trained models converged faster during the training, indicating that transfer learning facilitates fast convergence in the learning of pre-trained models (see **Figures 4A, C**).

The proposed technique for advancing diagnostic capabilities comprises two key stages. First (i.e., stage 1), a 26-layer CNN model was developed to detect dementia using MRI slices. Next (i.e., stage 2), the weights of the developed model were reutilized to subclassify the dementia class. In the first stage, to detect only dementia, the results showed that the proposed model yielded a high accuracy of 98.24% for binary classification, with a TPR of more than 98% for both classes (see **Figure 5A**). In the second stage, the developed CNN model was reused using the transfer learning concept for dementia subclassification. The results showed that only one sample out of 512 samples was misclassified, and the model produced a high classification rate of 99.7%, with a very high TPR and low FDR (see **Figure 5B**). Furthermore, fast convergence was observed as a result of transfer learning (**Figure 6B**). These results support our hypothesis that the frozen weight of a trained model from correlated images benefits transfer learning and results in a high classification performance. To further evaluate the performance of the model against data leakage issues, the authors have further performed 10-fold cross-validation. The proposed multistage framework shows almost similar high classification accuracy, further validating the effectiveness of the proposed approach (**Figure 7**). Another ADNI MRI scan dataset was used to validate the efficacy of the proposed framework. The framework validated the 100% classification rate of the developed CNN for dementia detection and subclassification (**Figure 8**). **Table 6** compares the proposed multistage framework with those of recent studies.

**Table 6:** Comparison of the proposed multistage CNN-based framework with recent research.

Study	Accuracy (%)	
	ADNI_Extracted _Axial dataset	Alzheimer’s Dataset
Wang et al. (41)	97.52	–
Mohammed et al. (42)	–	94.8
Acharya et al. (43)	–	95.70
El-Latif et al. (44)	–	95.93
Murugan et al. (29)	–	95.23 (augmentation)
Loddo et al. (45)	99.22	97.71
Kaplan et al. (46)	–	99.62 (10-fold) (healthy vs dementia)
Ching et al. (47)	98.93	–
Mohammad and Ahmadi (48)	99	–
Hasan and Wagler (49)	–	99.06
Shukla et al. (50)	–	99 (for dementia detection) 94 (dementia subclassification)
Latif et al. (44)	–	99.2 (for dementia detection) 95.93 (dementia subclassification)
Proposed multistage CNN-based framework	100	98.24 (for dementia detection) 99.70 (dementia subclassification)

CNN, convolutional neural network.

After deeply analyzing the results presented in **Table 6**, it can be concluded that the proposed CNN-based framework has the highest classification rate compared to others. These outcomes underscore the efficacy of the proposed model in efficiently and accurately handling the classification task, emphasizing its potential as a robust solution in AD detection and subclassification.

A single-modality dataset was used to evaluate the performance of the proposed network. In the future, multiple-modality datasets may be utilized to improve the classification performance for AD diagnosis. In addition, this study proposes a simple architecture; however, more intuitive architectures, such as transformers or the incorporation of attention networks, may be tested in the future. Confounding variables, such as the independent variable (imaging data in this case) and the dependent variable (presence or absence of AD), should be considered in designing AD studies. These confounding variables can introduce spurious correlations, leading to reduced AD identification accuracy. The key clinical confounding variables to be considered include age, education, vascular health, genetic factors, and lifestyle. For instance, a CNN model trained on an older-skewed dataset might learn age-related features instead of AD-specific ones, resulting in misdiagnosis for younger AD patients and overdiagnosis in healthy older adults. Other important confounding variables related to the imaging data of AD identification include data collection, preprocessing, model designing, and the evaluation of model performance for variables unseen during training. Image quality can be affected and data inconsistencies introduced by differences in acquisition paradigms, spatial resolution, and magnetic field strength. Furthermore, inadequate preprocessing can result in artifacts, spatial distortions, and data inconsistencies, all of which can have an impact on the accuracy and reliability of the study. Model architecture, hyperparameter tuning, and regularization techniques are also important in reducing confounding effects and maximizing model performance. Finally, the evaluation of the model must be done by employing a rigorous validation process. For example, *k*-fold cross-validation can assist in reducing the impact of data variability and produce more reliable estimates of model performance. Evaluation criteria should also be carefully chosen to take into consideration confounding variables unique to AD identification tasks, such as class imbalance and susceptibility to false positives.

## 5 Conclusion

AD is a common and devastating neurological condition that substantially reduces the quality of life in affected individuals. These effects affect not only patients but also their families and society. Timely diagnosis is critical to adequately control AD and reduce its socioeconomic consequences. This paper proposes a multistage CNN-based AD detection and subclassification framework. A 26-layer CNN model was developed from scratch using MRI images to detect dementia. The model yielded a high accuracy of 98.24% in dementia detection using an online AD dataset. Subsequently, the developed CNN model was reutilized for the subclassification of dementia classes using transfer learning. This yielded a high accuracy rate of 99.70%, with only one misclassified sample. Moreover, another AD dataset was used to validate the model, and the results showed a 100% performance rate. The proposed framework was also compared with various pre-trained models and the latest literature to prove the effectiveness and superiority of the proposed model.

## Data availability statement

The original contributions presented in the study are included in the article/supplementary material. Further inquiries can be directed to the corresponding author/s.

## Ethics statement

Ethical approval was not required for the study involving humans in accordance with the local legislation and institutional requirements. Written informed consent to participate in this study was not required from the participants or the participants’ legal guardians/next of kin in accordance with the national legislation and the institutional requirements.

## Author contributions

MA: Conceptualization, Methodology, Software, Writing – original draft. KK: Conceptualization, Methodology, Software, Writing – review & editing. MK: Conceptualization, Formal analysis, Writing – review & editing. MF: Formal analysis, Investigation, Writing – review & editing. AZ: Formal analysis, Investigation, Validation, Visualization, Writing – review & editing. SL: Funding acquisition, Project administration, Resources, Supervision, Writing – review & editing.
